# Editorial: Controversies in Pediatric Cardiology

**DOI:** 10.3389/fped.2020.00424

**Published:** 2020-07-28

**Authors:** Antonio F. Corno, David S. Crossland

**Affiliations:** ^1^McGovern Medical School, Houston Children's Heart Institute, Children's Memorial Hermann Hospital, University Texas Health, Houston, TX, United States; ^2^Adult Congenital and Pediatric Heart Unit, Freeman Hospital, Newcastle upon Tyne, United Kingdom; ^3^Cardiovascular Research Centre, Institute of Genetic Medicine, Newcastle University, Newcastle upon Tyne, United Kingdom

**Keywords:** cardiology, pediatric cardiology, cardiology controversies, editorial, pediatric

There remain many controversies across the spectrum of congenital cardiology which are not limited to rare diagnoses and uncommonly preformed procedures. Factors contributing to this include a paucity of randomized trials, the heterogeneous nature of congenital cardiac disease (even within each headline diagnosis), differences health care systems offering care and an enthusiasm to carry out new procedures, often related to morbidity and mortality associated with current strategies. A natural and useful way of addressing and discussing these controversies is as part of debate sessions at speciality meetings. Although these are often delivered by experts within that area the total audience is often limited to those attending the meeting. The purpose of this Research Topic was to extend this debate to print allowing review of more detailed argument and data presented by the experts to a wider audience.

The three topics considered in this Research Topic are the pulmonary valve replacement for pulmonary valve regurgitation, the current role of echocardiography in the diagnosis of congenital heart defects, and the best modality of cardiopulmonary bypass for pediatric heart surgery.

In developed health care systems pulmonary valve replacement is the third most common procedure in congenital cardiology after atrial septal defect closure and patent ductus arteriosus closure. Between 2014 and 2017 1,039 such procedures were carried out in the UK (260 in children and 779 in adults) of which 803 were surgical and 260 transluminal with a reported 30 day mortality for either procedure of <1%^1^. Given that this is a high frequency low mortality procedure debate as to the most appropriate option must also explore morbidity and long term outcomes from the procedure. In this edition Morgan and Corno present the controversy surrounding whether pulmonary valve regurgitation should be addressed surgically or interventionally from the point of view of the interventional cardiologist and the cardiac surgeon.

Morgan explores the rapid developments in both the technique and technology in the field of percutaneous pulmonary valve implantation. Previous perceived limitations of the procedure and how these are being addressed are explored. In particular techniques used in the presence of small right ventricular outflow tracts, including hybrid techniques, and large outflow tracts, including stents to build landing zones for valve implantation, developments of valve technology to fill the redundant space, and use of a branch pulmonary artery to secure the some types of valve. The latest available models of percutaneously implantable valves are described in detail, with potential advantages and disadvantages to orient the readers in the choice (Morgan). Finally in the discussion the potential drawbacks and complications are thoroughly analyzed. Specific procedural complications are discussed including the possibility of conduit rupture, valve embolization, tricuspid valve damage, and coronary compression as well as how these can be avoided to give a very low procedural mortality and morbidity. One of the main concerns about percutaneous valves, namely the incidence of endocarditis is discussed, with the suggestion that much of this may be related to the (often necessary) use of bovine jugular grafts, however these are placed, and that this will remain a hot topic going forward. The paucity of high quality long-term data that can be used to directly compare percutaneously and surgically implanted valves is highlighted as a major limitation to comparing the two options, in particular the using “freedom from death or re-intervention” as a the main comparator.

Corno explores all the currently available options to implant a pulmonary valve either with direct surgical technique as well as hybrid approach combining surgical exposure and percutaneous type of valve implantation, with pros and cons of both types of choice. The interventionalists might argue that this middle ground approach demonstrates increasing surgical acceptance of the percutaneous option! He considers in the decision-making process the following elements: (a) morphology and size of the right ventricular outflow tract, in particular in the presence of a previously implanted trans-annular patch; (b) morphology and size of the pulmonary arteries in the case a required enlargement or reconstruction; (c) presence of residual intra-cardiac defects; (d) presence of extremely dilated and dysfunctional right ventricle. Clearly the definitive choice will depend upon the match of the characteristics of the individual patient with the experience and outcomes of the operators. Each caregiver has to keep in mind that this type of problem, pulmonary valve regurgitation, exposes each patient to repeated procedures during the rest of his/her life, and therefore the choice has to take in account the options left available for the future needs (Corno).

With regard to the topic “is echocardiography enough or cath-angio/CT scan/MRI are still needed?,” the group of Márquez-González et al. reported a very large study of patients in Mexico with right ventricular outflow tract obstruction. They compared the non-invasive approach using the echocardiogram derived Tei index (defined as the ratio of isovolumetric contraction time to ejection time) with an invasive cardiac catheterization definition of right ventricular dysfunction. The authors provide interesting data that the Tei index in this group of patients can accurately predict catheterization-based diagnosis of right ventricular dysfunction (Márquez-González et al.). Although the limitations of a lack of comparison with MRI and that catherization-based right ventricular dysfunction is not synonymous with the need for intervention, the inferences of this study may be important for low and middle-income environments where resources are not always available to perform other imaging modalities.

Finally Corno et al. reported a clinical study with comparison between two groups of patients homogeneous for age and weight, with the same STAT risk stratification, operated on for congenital heart defects, one with normothermic and the other with mild hypothermic cardiopulmonary bypass. The data collected in the operating room and during the first 24 post-operative hours shows that the normothermic group have a favorable difference in biomarkers (arterial PH, lactate), lower vasoactive inotropic score, reduced chest drain losses, reduced transfusion requirement, shorter duration of mechanical ventilation and shorter stay in Pediatric Intensive Care Unit (PICU). In addition to the improved immediate clinical outcomes, the normothermic group had lower average cost per patient day of stay in PICU (Corno et al.). The authors conclude that wider use of normothermic cardiopulmonary bypass should be taken into consideration for pediatric cardiac surgery and suggest prospective multi-center randomized controlled trials in a risk adjusted population of patients with complex congenital heart defects to support their results.

## Author Contributions

AC drafted the manuscript. All authors revised and approved the final version.

## Conflict of Interest

The authors declare that the research was conducted in the absence of any commercial or financial relationships that could be construed as a potential conflict of interest.

## Footnotes

^1^https://nicor4.nicor.org.uk/CHD/an_paeds.nsf/vwContent/home (accessed April 28, 2020).

